# THE LINK BETWEEN VIOLENCE EXPOSURE AND BIOLOGICAL AGING IN MID-LIFE MUSLIMS IN ISRAEL

**DOI:** 10.1093/geroni/igad104.0915

**Published:** 2023-12-21

**Authors:** Khalil Iktilat, Maayan Agmon

**Affiliations:** University of Haifa, Haifa, HaZafon, Israel; University of Haifa, Haifa, HaZafon, Israel

## Abstract

Trauma and accumulated chronic stress throughout life are associated with negative health outcomes; more specifically, exposure to violence has been linked to an accelerated aging state. In Israel’s Arab sector, rates of violence have been steadily rising for the past several years. Homicide rates, relative to population size, are seven times higher among Arabs. We aimed to determine the effects of exposure to violence on estimation of biological age as a measure of rate of aging within the ethnic minority of Muslims in Israel. We have conducted a cross-sectional study that included 375 participants age 50.5±6.9 years, of which 61% women. Biological age was estimated using the Klemera-Doubal method and physiological biomarkers; exposure to violence was measured using the Screen for Adolescent Violence Exposure questionnaire followed by a principal component analysis. The average total score of violence exposure was 1.83±0.36 (range 1-5, 1 corresponds with lower exposure). We found a positive and significant relationship among biological age, the “direct evidence of violence” component, and the “evidence of severe violence in the immediate environment” component (r = 0.119, p<0.05; r = 0.111, p<0.05, respectively). These results demonstrate that exposure to violence negatively affects health and accelerates aging within Israel’s Muslim minority. Comprehensive, culturally- and gender-adapted programs are required to reduce violence and address its contribution to the negative aging trajectory in this population.

